# Distinguishing Low and High Water Consumers—A Paradigm of Disease Risk

**DOI:** 10.3390/nu12030858

**Published:** 2020-03-23

**Authors:** Lawrence E. Armstrong, Colleen X. Muñoz, Elizabeth M. Armstrong

**Affiliations:** 1 Professor Emeritus, Human Performance Laboratory and Department of Nutritional Sciences, University of Connecticut, Storrs, CT 06269, USA; 2 Assistant Professor, Department of Health Sciences, University of Hartford, West Hartford, CT 06117, USA; cmunoz@hartford.edu; 3 Registered Dietitian, Riverside Behavioral Health Center, Hampton, VA 23666, USA; elizabeth.armstrong@rivhs.com

**Keywords:** arginine vasopressin, cortisol, plasma osmolality, dietary protein, dietary salt, thirst

## Abstract

A long-standing body of clinical observations associates low 24-h total water intake (TWI = water + beverages + food moisture) with acute renal disorders such as kidney stones and urinary tract infections. These findings prompted observational studies and experimental interventions comparing habitual low volume (LOW) and high volume (HIGH) drinkers. Investigators have learned that the TWI of LOW and HIGH differ by 1–2 L·d^−1^, their hematological values (e.g., plasma osmolality, plasma sodium) are similar and lie within the laboratory reference ranges of healthy adults and both groups appear to successfully maintain water-electrolyte homeostasis. However, LOW differs from HIGH in urinary biomarkers (e.g., reduced urine volume and increased osmolality or specific gravity), as well as higher plasma concentrations of arginine vasopressin (AVP) and cortisol. Further, evidence suggests that both a low daily TWI and/or elevated plasma AVP influence the development and progression of metabolic syndrome, diabetes, obesity, chronic kidney disease, hypertension and cardiovascular disease. Based on these studies, we propose a theory of increased disease risk in LOW that involves chronic release of fluid-electrolyte (i.e., AVP) and stress (i.e., cortisol) hormones. This narrative review describes small but important differences between LOW and HIGH, advises future investigations and provides practical dietary recommendations for LOW that are intended to decrease their risk of chronic diseases.

## 1. Introduction

Water is essential for digestion, circulation of nutrients, movement of substances across cell membranes, metabolism and regulation of intracellular-extracellular concentration. These processes are optimized by the stringent central nervous system defense of body water and fluid concentration. Changes of body water volume and osmolality are monitored by the brain, renal water and electrolyte excretion/retention is regulated by neuroendocrine responses and fluid-electrolyte movements between intracellular and extracellular fluid compartments maintain equilibrium [1]. These complex, dynamic processes act to regulate body water and plasma osmolality (P_OSM_) within 1% of baseline, from one day to the next [2,3]. The primary regulatory components of fluid-electrolyte homeostasis are thirst (i.e., an aversive state that motivates water-seeking and drinking), pituitary release of arginine vasopressin into the circulation (AVP; a hormone that acts to regulate total body water and extracellular concentration by modulating water excretion at renal nephrons, constricting blood vessels and preserving blood pressure); increased plasma angiotensin II (ANG II; regulates blood volume and pressure; renal sodium retention as part of the renin-angiotensin-aldosterone system (RAAS); stimulates thirst and AVP release); oropharyngeal afferent signals that modulates thirst, drinking, plasma AVP and plasma ANG II [1,4] and release of atrial natriuretic peptide (ANP; a hormone released in response to atrial stretch receptors) and apelin (a hormone that opposes the actions of AVP; [5,6]) to remediate plasma volume expansion and dilution.

Most humans do not experience severe dehydration (> 5% body weight loss) during ordinary daily activities but reach a state of mild dehydration (1–2% of body weight lost as water) multiple times each week. These individuals are rarely aware of mild dehydration because thirst, the only easily-recognized symptom, is not perceived until 1–2% of body weight is lost [3]. Unfortunately, for reasons that have not been clarified, some individuals habitually consume little water each day (LOW, low volume drinkers). For example, 25–33% of all adults in the United States and Europe consume less than 1.5 L of water per day (i.e., total water intake (TWI) = plain water + beverages + food moisture) [7,8,9]. This volume is considerably less than the Adequate Intakes for water recommended by the European Food Safety Authority [10] and the U.S. National Academy of Medicine [11] for men (2.5,3.7 L·d^−1^) and for women (2.0,2.7 L·d^−1^). The TWI of LOW also are considerably lower than the volumes that are prescribed by physicians [12] to reduce the risk of chronic kidney disease [13,14], the recurrence of kidney stones [15] and urinary tract infections [16]. Interestingly, despite regularly consuming low volumes of water, these individuals do not appear to demonstrate the expected counterregulatory increase in thirst and may even experience a weaker thirst signal than individuals who regularly consume higher fluid volumes [17,18].

At the beginning of this century, researchers reported that mild dehydration played a role in the development of various acute and chronic diseases and that improved hydration status positively affected diseases of the cardiovascular system, eyes and oral cavity [19]. Subsequently, while investigating the possibility that a low TWI is associated with an increased risk of degenerative diseases or diminished physiological function, investigators began to compare numerous water-relevant variables of LOW to those of habitual high volume drinkers (HIGH), including perceptual ratings (e.g., thirst sensation using a visual analog scale or category numerical ratings) and plasma AVP concentrations. In these observational studies [9,18,20,21], the 24-h TWI of LOW ranged from 0.7–1.6 L·d^−1^ and HIGH ranged from 2.5– 3.3 L·d^−1^. Urinary variables (e.g., urine specific gravity (U_SG_), osmolality (U_OSM_), color (U_COL_) and volume (U_VOL_)) distinguished LOW from HIGH, in that LOW excreted a smaller volume of concentrated urine because they consumed less water each day [9,18]. 

Although the acute effects of moderate-to-severe dehydration have been extensively studied for over 70 years [22,23,24,25], investigators knew little about LOW prior to 2008 because research reports regarding the consequences of chronic mild dehydration were rare [26,27]. Between the years 1996 and 2003, animal experiments and clinical studies revealed the adverse effects of elevated AVP on kidney diseases, albuminuria and hypertension [28]. Then, when physiologists discovered that both LOW and HIGH exhibited a normal P_OSM_ and body weight (i.e., suggesting euhydration) but that LOW had higher plasma AVP compared to HIGH, they began to ask, “What are the possible mechanisms of long-term negative health effects that result from habitually consuming a small volume of water?”. This prompted human epidemiological studies which eventually revealed statistically significant links between elevation of plasma AVP or copeptin (i.e., released in equimolar quantities with AVP) and increased risk of developing diabetes [29,30,31], hyperglycemia [32], insulin resistance, metabolic syndrome [33,34], abdominal obesity [35], stroke, cardiovascular disease, cardiovascular events, cardiovascular death [7,36,37,38], hypertension [34] and kidney disease [38,39,40]. In some of these studies, the data of thousands of adults were involved in the analysis, providing robust statistical power. Although a number of factors stimulate the release of AVP [41], investigators recommend increased daily water intake as a safe, cost-effective, simple preventive intervention to lower plasma AVP [7,42]. Controlled laboratory animal experiments also support this recommendation, in that the increased daily water consumption of obese Zucker rats, which reduced circulating levels of plasma AVP, was associated with a reduced incidence of liver steatosis [43].

In consideration of the above, the purposes of the present review are fourfold. First, to describe human hydration as a complex, dynamic continuum that is primarily but not exclusively influenced by small changes in AVP release in response to differing levels of water intake. Second, to review the physiological, perceptual and behavioral characteristics that distinguish LOW from HIGH. Third, to describe a plausible mechanism of increased disease risk for LOW that involves the chronic release of fluid-electrolyte and/or stress hormones. Fourth, to propose dietary and hydration recommendations for LOW that encourage increased daily TWI and decrease plasma AVP concentration. 

## 2. The Hydration Continuum

**Figure 1:**
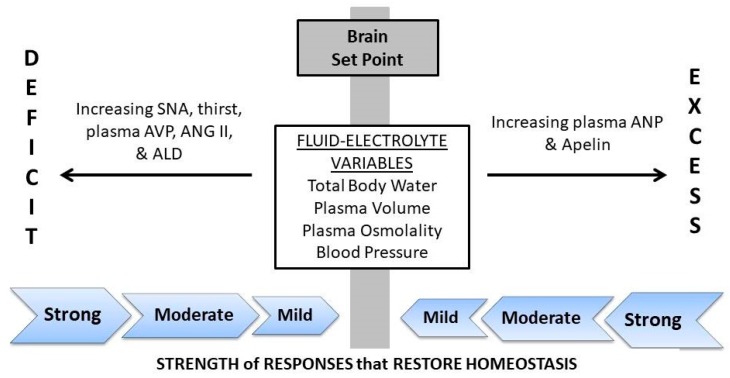
The hydration continuum. Perturbations of fluid-electrolyte variables initiate thirst and neuroendocrine responses to restore homeostasis and to maintain optimal health and function. The magnitude of a fluid-electrolyte deficit (DEFICIT) or fluid-electrolyte excess (EXCESS) determines the strength of responses that return volume, osmolality and pressure back to the encoded brain set point. Abbreviations: SNA, sympathetic nerve activity; AVP, arginine vasopressin; ANG II, angiotensin II; ALD, aldosterone; ANP, atrial natriuretic peptide. Revised from Armstrong & Johnson, 2018 [41].

A recent editorial by Kavouras [44] proposed the novel term “underhydration” to describe LOW as individuals who (a) exhibit normal P_OSM_ and body water; (b) present with elevated plasma AVP and concentrated urine biomarkers; (c) consume low volumes of water daily while counterintuitively exhibiting a low thirst rating. This description serves as a reminder that it is difficult to define and describe a complex, dynamic process such as human fluid-electrolyte regulation [45] in a way that is accurate and valid for all situations [46]. Because total body water and extracellular concentration fluctuate continuously throughout life, precise definitions for terms such as dehydration, hypohydration and euhydration are inadequate. Thus, in the present review, we utilize a graphic illustration of the hydration continuum (i.e., a complex, dynamic process) that is compatible with the known characteristics of LOW and HIGH (Figure 1; modified from Reference [41]). The central vertical gray zone of this figure represents the brain set point (i.e., a narrow encoded range in which the body functions optimally) that is unique to each of the four regulated fluid-electrolyte variables (center rectangle). The regions to the left and right of the set point zone represent a water or sodium deficit or excess; the two thin black arrows, which point away from the set point, illustrate a deficit or excess of water or sodium. Because both LOW and HIGH exist near the set point zone during daily activities, many but not all of their physiologically-regulated variables are similar. The extreme left and right sides of Figure 1 represent the greatest perturbations of each regulated variable due to severe dehydration, overdrinking, a large dietary osmotic load and a large sodium loss in sweat and/or urine. The shaded block arrows which point toward the central set point (i.e., labeled strong, moderate, mild) illustrate the strength of neuroendocrine responses which move each regulated variable toward the set point, in an effort to restore altered homeostasis. If all fluid-electrolyte variables are at or near the set point zone, a state of euhydration exists because the brain is activating minor or no compensatory responses (e.g., low or basal plasma AVP or ANG II). LOW individuals, however, are distinct from HIGH because they have elevated plasma AVP and excrete a small volume of concentrated urine.

## 3. The AVP-TWI Relationship

The knowledge that LOW and HIGH have different circulating AVP concentrations and urinary biomarkers (i.e., U_VOL_, U_OSM_, U_SG_) previously was considered to be unimportant because both LOW and HIGH exhibited numerous characteristics which fell within the reference ranges of laboratory values for healthy adults (Table 1). 

**Figure 2:**
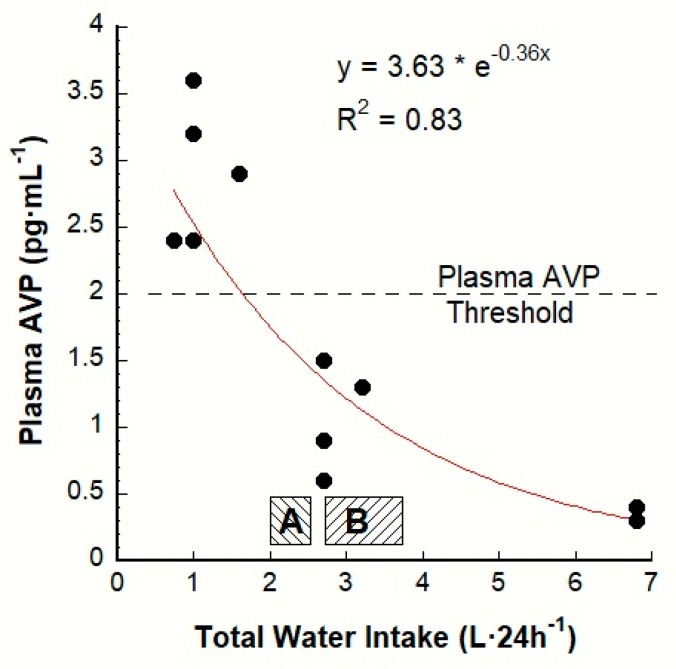
The relationship between daily total water intake (all sources) and plasma AVP concentration, based on 11 published laboratory investigations. The plasma AVP threshold of 2 pg·mL^−1^ [41] occurs at a TWI of 1.8 L·24 h^−1^, which is approximately 64 total oz of water per day. Low volume drinkers and water-deprived adults appear as the 5 data points above the plasma AVP threshold. See text for details. The range of adequate intakes for women and men appear as zone A (European Food Safety Authority [10]) and zone B (U.S. National Academy of Medicine [11]).

This perspective is evolving today, as described in the Introduction section above, because a growing body of epidemiological evidence consistently shows statistically significant relationships between elevated plasma AVP and increased risk of degenerative diseases. Thus, we analyzed previous human research [9,18,50] to determine the approximate TWI at which plasma AVP increases. A graphic representation of the AVP-TWI relationship appears in Figure 2 (modified from Reference [41]); each data point is a group mean value, measured once per day. The statistically determined plasma AVP threshold (2 pg·mL^−1^) occurs at a TWI of 1.8 L·24 h^−1^ (i.e., approximately 64 oz of total water per day) and represents the encoded set point at which the brain initiates the AVP neuroendocrine response to conserve water (see Figure 1); this TWI threshold for AVP release likely would not change due to sweat losses (i.e., during exposure to a hot environment or prolonged physical activity) but the total daily water requirement would increase for that day. Regulated variables each have a unique set point that optimizes whole-body fluid-electrolyte balance. Although 2 pg·mL^−1^ is not acknowledged as a human plasma AVP threshold by other authors, published figures and tables [3,51,52,53,54,55,56,57,58] support this paradigm. Additional support appears in Table 1, which shows that the mean plasma AVP levels of LOW and HIGH lie immediately above and below (i.e., virtually equidistant from) a plasma AVP concentration of 2 pg·mL^−1^. Verification of this 2 pg·mL^−1^ AVP threshold or discovery of a mean value for healthy humans, could be accomplished by testing plasma AVP or copeptin concentration (i.e., which is released in equimolar quantities with AVP) of individuals who consume less than and more than 1.8 L·24 h^−1^. Further, the influence of environmental conditions (e.g., cold or hot ambient temperatures), age or sex on the plasma AVP threshold could be evaluated within a repeated measures experimental design or by comparing groups of men and women across a range of ages.

The exponential regression line of best fit in Figure 2 represents central nervous system integration of numerous afferent signals (i.e., blood volume, concentration, pressure) across a range of TWI from 0.7 to 6.8 L·24 h^−1^. This relationship exists primarily because of the chain of events involving water consumption, P_OSM_ and plasma AVP in that water consumption dilutes blood, reduces P_OSM_ and decreases plasma AVP. During ordinary daily activities (i.e., when circulatory stress and homeostatic perturbations are minor), the relationship between P_OSM_ and plasma AVP is linear and strongly correlated [59]; when P_OSM_ changes are pronounced (e.g., the TWI extremes shown in Figure 2), a curvilinear model produces a better fit [60].

The two zones that appear directly above the horizontal axis of Figure 2 illustrate the range of adult water Adequate Intakes recommended by the European Food Safety Authority (zone A; women 2.0 and men 2.5 L·24 h^−1^; [10]) and the U.S. National Academy of Medicine (zone B; women 2.7 and men 3.7 L·24 h^−1^; [11]). These Adequate Intakes (zones A and B) result in basal levels of plasma AVP below the 2.0 pg·mL^−1^ threshold. Low volume drinkers appear in Figure 2 as the 5 data points above the plasma AVP threshold; these values (2.4–3.6 pg·mL^-1^) imply a continuous, elevated release of AVP under conditions of euhydration (i.e., a P_OSM_ within the laboratory reference range, see Table 1, column 5). 

## 4. Characteristics that Distinguish Low from High

### 4.1. Longitudinal Clinical Trials

Unfortunately, it is difficult to conduct long-term randomized clinical trials that evaluate associations between chronic low water intake and development of degenerative diseases or reduced longevity, for the following three reasons [27]. First, chronic diseases often develop across years or decades and intervention studies suffer from participant noncompliance and attrition; it is difficult for any person to maintain a constant hydration state across many years of life. Second, the number of subjects required for adequate statistical power is large and research is costly. Third, multiple personal characteristics, dietary habits or lifestyle behaviors may concurrently encourage disease development, with their intercorrelation making interpretation of a single factor (e.g., daily water intake) difficult. 

### 4.2. Observational Studies and Controlled Interventions

Table 1 presents a comparison of blood and urine characteristics of healthy LOW and HIGH individuals, as published in four previous investigations. These values provide a baseline comparison of the physiological state of LOW versus HIGH. Importantly, all values in Table 1 fall within laboratory reference ranges of healthy adults (column 5), including P_OSM_ and S_OSM_. Between-group comparisons also indicate no statistical differences (*p* > 0.05) of extracellular osmolality (P_OSM_ and S_OSM_) and serum sodium (S_Na+_), although the mean LOW values consistently tend to be greater than those of HIGH. In classical terms, these findings suggest that both LOW and HIGH live in a state of euhydration (e.g., within the set point zone in Figure 1; [2,11,21]), even though their 24-h water intakes differ by 1.5–2.1 L·24 h^−1^ (Table 1). In contrast to these non-significant findings, urine biomarkers (U_VOL_, U_OSM_, U_SG_) indicate statistically significant between-group differences (range, *p* < 0.05 to 0.001), with LOW exhibiting a smaller 24-h urine volume and a more concentrated urine (versus HIGH). Because the urinary characteristics of LOW and HIGH are the products of sensitive and precise regulation of body fluid concentration and volume [1,61], they are relevant to the P_OSM_, S_OSM_ and P_AVP_ findings in Table 1. Interestingly, AVP is likely to be the hormone primarily responsible for between-group differences in the maintenance of body water and concentration, in that publications have reported no LOW versus HIGH differences of plasma renin [9] and aldosterone [9,18] concentrations.

Inherently, LOW individuals habitually consume less water than HIGH each day. One simple explanation for this phenomenon involves the sensation of thirst and motivation to drink. In fact, two published investigations [17,18] have reported a LOW (0.7–1.6 L·d^−1^) versus HIGH (2.7–3.2 L·d^–1^) difference of thirst intensity, measured in France (visual analog scale, 11 ♂, 41 ♀) and in the USA (9 category rating scale, 28 ♀). In both studies, LOW reported significantly less intense thirst (e.g., 66–77% of HIGH ratings; *p* < 0.001 and *p* = 0.002, respectively). This difference existed in one study when both groups consumed a smaller volume (1.6–2.0 L·d^−1^) and a larger volume (3.2–3.5 L·d^−1^) for 4 d [18] and suggests that indeed a reduced thirst intensity is one reason that LOW individuals live chronically with a mildly elevated plasma AVP. This concept is discussed further in Section 7.2 below. With regard to the motivation to drink, no controlled experiments have compared the motivation levels of LOW and HIGH consumers, to our knowledge. 

Three investigations have intervened in the water homeostasis of LOW and HIGH by reversing their habitual 24-h water intakes. Table 2 presents the findings of these investigations [18,20,21] in the form of physiological changes from baseline that occurred across 3–4 d when LOW consumed 1.5–1.9 L·d^−1^ more than usual and when HIGH decreased their usual intake by 1.2–1.6 L·d^−1^. As anticipated, P_AVP_ decreased and U_VOL_ increased in the modified LOW group, whereas these responses changed in the opposite directions in the modified HIGH group. The outcome of modified 24-h TWI (Table 2) was that blood concentration as a primary regulated variable was statistically similar for LOW and HIGH (*p* > 0.05, NS) and changes of P_OSM_ and S_OSM_ were small (0 to −2 mOsm·kg^−1^ for LOW; +1 to +3 for HIGH).

**Figure 3:**
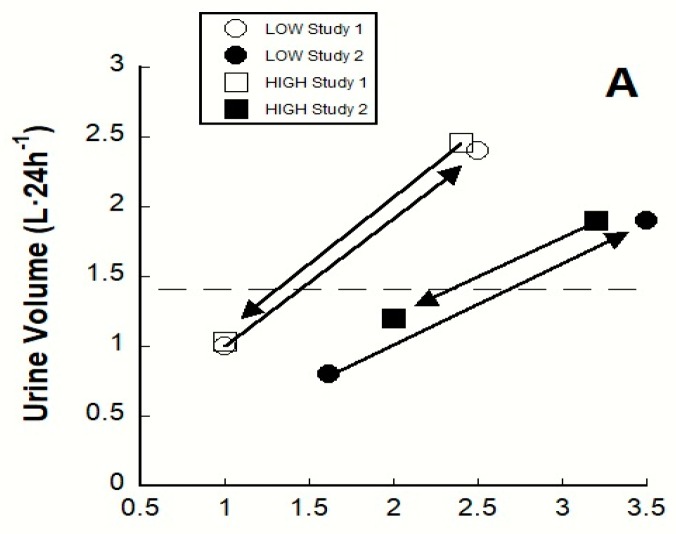

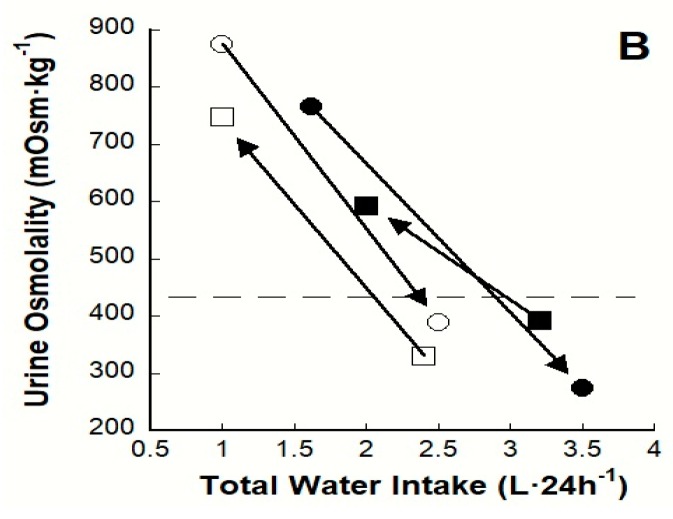
Relationships of total water intake to urine volume (panel **A**) and urine osmolality (panel **B**), when low volume drinkers (LOW, *n* = 14–30 each symbol) and high volume drinkers (HIGH, *n* = 14–22) reversed their habitual 24-h TWI during two investigations. Open symbols represent study 1 [20] and closed symbols represent study 2 [21]. Arrows denote changes from baseline (pre-intervention) to day 3 or 4 of each modified TWI. The horizontal dashed line in Panel **A** and Panel **B** represent the urine values that exist when plasma AVP is 2.0 pg·mL^−1^. Experimental design details appear in the footnotes of Table 1 and Table 2.

Figure 3 graphically illustrates the direction of these within-group changes as arrows extending from baseline. Details of the experimental designs (study 1 and study 2) appear in Table 1. The slopes (i.e., gain) of the arrows for U_OSM_ and U_VOL_ are similar in both published studies [20,21], suggesting that the relationship between TWI and U_OSM_ in panel A, as well as that between 24-h TWI and U_VOL_ in panel B (i.e., both of which represent fluid-electrolyte regulation), were similarly sensitive regardless of the initial habitual fluid intake (LOW or HIGH) or whether TWI increased or decreased during experimental interventions.

## 5. Possible Mechanisms of Morbidity and Mortality: AVP and Cortisol

One salient theory [7] recognizes that an elevated plasma AVP concentration participates in the body’s response to stress by stimulating adrenocorticotrophic hormone (ACTH) release (i.e., initiating activity along the hypothalamic-pituitary-adrenal axis; HPA). In fact, AVP [62,63] and copeptin (i.e., released in equimolar quantities with AVP) [64,65] often are considered to be stress hormones. ACTH release also is stimulated by corticotropin-releasing factor (CRF). AVP potentiates the effect of CRF and, working together, these two agents are the primary promoters of ACTH secretion [64]. ACTH, in turn, stimulates the adrenal cortex to produce cortisol, a glucocorticoid hormone and widely-recognized plasma biomarker of stress [64]. Cortisol exerts inhibitory feedback on CRF at the hypothalamus and on ACTH secretion in the anterior pituitary. Although AVP stimulation of ACTH is not responsive to cortisol feedback [66], cortisol can directly influence body water and extracellular concentration by increasing urine volume, urinary sodium excretion and potassium excretion; these effects may result in negative whole-body water balance [67]. Further complicating these neuroendocrine responses, men fall into two groups of differential HPA axis sensitivity (i.e., high responders and low responders, based on plasma ACTH and cortisol concentrations) during both psychological and exercise stress [63]. This suggests that the adrenal cortexes of high responders are hypertrophic and/or hypersensitive to ACTH and represents an important source of inter-individual variability.

Prolonged periods of heightened HPA activity with elevated plasma cortisol can be deleterious to health and longevity. For example, Cushing’s Syndrome is a severe, chronic, systemic condition that results from glucocorticoid (e.g., cortisol) excess. Patients with this illness have increased morbidity and mortality that often includes the following: visceral obesity, dyslipidemia (e.g., high triacylglycerols and low HDL-cholesterol levels), arterial hypertension, cardiovascular abnormalities and disordered body fluid homeostasis [68,69]. Elevated plasma cortisol (i.e., HPA axis hyperactivity) also has been theoretically associated with the pathologies of insulin resistance, hyperglycemia, metabolic syndrome [70] and subtypes of major depression [66,71]. This latter finding is compatible with four previous observational studies in which 24-h TWI was associated with differences of self-reported mood states. Using a validated test instrument, LOW reported greater tension, depression, confusion and fatigue, as well as less vigor [8,17,72,73]; unfortunately, none of these studies measured plasma cortisol. Hyperactivity of the HPA axis also has been theoretically implicated in premature aging. Known as the Glucorticoid Cascade Hypothesis, this concept emphasizes impairment (i.e., of terminating the release of neuroendocrine hormones at the end of a stressful event) and slowing of responses that counteract mild sustained stress [74]. This paradigm proposes that hypersecretion results from age-related degeneration of hippocampal neurons that normally inhibit glucocorticoid release; this degeneration purportedly results from cumulative exposure to cortisol and other stress hormones.

Animal research points to AVP as a contributor to the release of ACTH and cortisol during some but not all types of *acute* stress, with CRF as the primary regulator [75]. In contrast, during repetitive *chronic* stress, evidence demonstrates that regulation of ACTH and cortisol switches from CRF to AVP (i.e., concurrent with upregulation of AVP receptors in the pituitary), suggesting that AVP has a primary role in HPA adaptation to long-term stress stimulation [75]. Indeed, human experiments show that a habitual low TWI (e.g., with plasma AVP > 2 pg·mL^−1^) influences plasma cortisol, as reported by Perrier and colleagues [9] who compared groups of LOW (TWI of 1.3 L·24 h^–1^, *n* = 39) and HIGH (3.4 L·24 h^−1^, *n* = 32). The LOW group exhibited a significantly greater (*p* = 0.01) plasma cortisol concentration, measured on three consecutive mornings. Further evidence in humans is found in a small interventional study (*n* = 5 males) which demonstrated a progressive reduction in salivary cortisol in response to a progressive increase of water intake, followed by a tendency for salivary cortisol to increase when water intake was subsequently reduced [76].

Regarding cardiovascular disease, evidence from human and animal research suggests that AVP has pro-atherogenic effects (e.g., encourages platelet aggregation and formation of fatty plaque in the arteries) [38]. With regard to type 2 diabetes, AVP may directly stimulate metabolism including hepatic glucose production [77] or insulin release from the pancreas [78] and may adversely affect whole-body responses to insulin [79]. Further, increased activity of the AVP system has been recognized as a unifying factor in the metabolic syndrome with potential importance in the treatment of cardiovascular disease [34].

It is relevant that men maintain a higher plasma AVP concentration than women. This was revealed in the experiments of Stachenfeld and colleagues [80] in which both men and women began with an identical P_OSM_ (286 mOsm·kg^−1^) and similar baseline plasma AVP concentrations (♀, 1.7; ♂, 2.2 pg·mL^−1^; compare to Figure 2), then received intravenous hypertonic saline (3% sodium chloride, NaCl). This infusion raised the peak P_OSM_ identically in both groups to 299 mOsm·kg^−1^ at 120 min, after which the plasma AVP of women and men peaked at 3.3 and 4.7 pg·mL^−1^, respectively. This likely explains why men inherently have an average urine osmolality that is 21%–39% greater than that of women. Because maximal urine concentration of ~1200 mOsm·kg^−1^ occurs when plasma AVP reaches 3–5 pg·mL^−1^ [81] (compare to Figure 2), this inherent male characteristic increases the risk of chronic kidney disease, kidney stones and hypertension—all factors that are related to renal concentration [82]. Additional considerations of renal osmolar excretion appear below in Section 7.3.

## 6. A Theoretical Paradigm of Disease Risk

**Figure 4:**
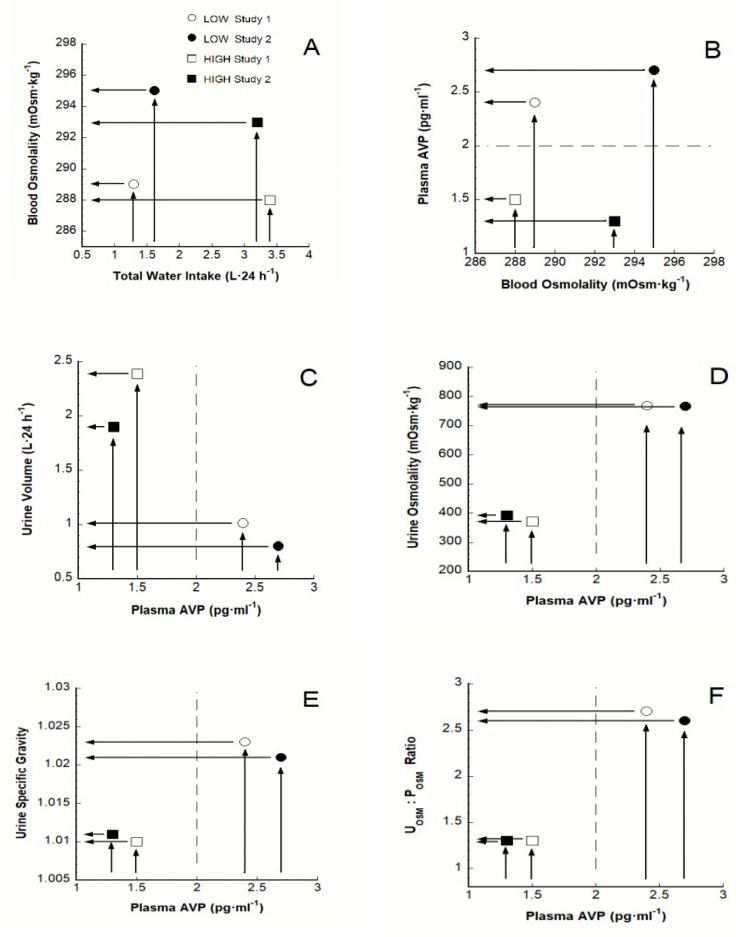
Relationships among total water intake, osmolality, AVP and four hydration biomarkers of LOW (○●) and HIGH (□■). A small blood osmolality difference (LOW versus HIGH; panels **A** and **B**) results in the subsequent responses illustrated in panels **B–F**. The plasma AVP threshold of 2 pg·ml^-1^ (see Section 3 above) is depicted as a dashed line in panels **B-F**. Open symbols represent study 1 [20] and closed symbols represent study 2 [21] baseline mean values (see columns 2 and 3 of Table 1). Arrows aid visual discrimination of LOW and HIGH values in studies 1 and 2. The blood osmolality reference range of laboratory values for healthy adults (panels A and B) is 285–295 mOsm·kg^−1^ [47]. (**A**): the influence of TWI on blood osmolality; (**B**): the influence of blood osmolality on plasma AVP concentration; (**C**): the influence of plasma AVP on urine volume; (**D**): the influence of plasma AVP on urine osmolality; (**E**): the influence of plasma AVP on urine specific gravity; (**F**): the influence of plasma AVP on the ratio of urine osmolality-to-plasma osmolality.

In this section, we review relevant published data and present a theoretical mechanism that differentiates LOW from HIGH. Figure 4 graphically depicts baseline mean values from Table 1. This figure illustrates the processes underlying the elevated plasma AVP of LOW and the resulting changes in urinary biomarkers. Beginning with panel A, both study 1 and study 2 [20,21] show that the mean baseline P_OSM_ of LOW is slightly greater (e.g., 1–2 mOsm·kg^−1^) than HIGH; this trend, albeit not statistically different, exists for all P_OSM_, S_OSM_ and S_Na+_ values in Table 1. This phenomenon was reported previously in humans whose daily water intake was rigorously controlled during laboratory experiments [20,50]. Intuitively, it may seem that such a small concentration difference, in a blood volume of ~5 L and an extracellular volume of ~14 L, would not affect physiological responses meaningfully, especially considering that all osmolality values in panel A are within the laboratory reference range (285–295 mOsm·kg^−1^) for healthy adults. But, quite to the contrary, a mean P_OSM_ difference between LOW and HIGH of only 0.3–0.7% (1–2 mOsm·kg^−1^ in panel B) resulted in a 60–108% (0.9–1.4 pg·mL^−1^) between-group difference in plasma AVP. This observation supports previously published human experiments [83]. Further, this small POSM difference (i.e., due to different TWI) positioned HIGH below the 2 pg·mL^−1^ plasma AVP threshold (see horizontal dashed line at Figure 4, panel B and Figure 2); in contrast, LOW is positioned above this threshold, suggesting that they may experience a continuously elevated release of AVP. 

Thus, we propose that the extraordinary sensitivity of AVP release from the posterior pituitary, in response to a small P_OSM_ change, is the mechanism that stimulates chronic HPA axis hyperactivity (see above) and distinguishes LOW from HIGH in terms of disease risk. Precise osmotic regulation of plasma AVP was first reported in dogs [84] and subsequently in humans over 40 years ago [61,85] as a means to maintain P_OSM_ within a narrow range (Figure 4, panels A and B). These insights explain the previously published observations that (a) the thirst threshold provides backup against inordinate decreases of total body water when the urinary compensatory capacity of AVP is maximized [58,61] and (b) urine volume is reduced 10- to 20-fold at the thirst threshold [86].

The precise sensitivity of plasma AVP regulation has important physiological implications for the identification of persons who habitually consume a low 24-h TWI. Panels C-E in Figure 4 depict the effects of AVP on reabsorption of water at renal collecting ducts. In concert, these mean baseline values show that decreased volume and increased concentration are characteristic of LOW. When considered in terms of U_OSM_ > 500 mOsm·kg^−1^, Table 1 also shows that HIGH consumed an adequate TWI (2.5–3.2 L·24 h^−1^); this urine concentration has been identified as a threshold that avoids an elevated plasma AVP and ensures urinary output sufficient to reduce the risk of kidney stone formation and renal function decline [87]. In contrast, LOW consumed an inadequate TWI, based on a U_OSM_ of approximately > 800 mOsm·kg^–1^ (Table 1); this urine concentration corresponds to inadequate 24-h TWI [88]. As such, urinary measurements can be used as biomarkers to distinguish LOW from HIGH and during self-assessment of hydration status [87]; the latter procedure is described below in Section 8.5.

**Figure 5:**
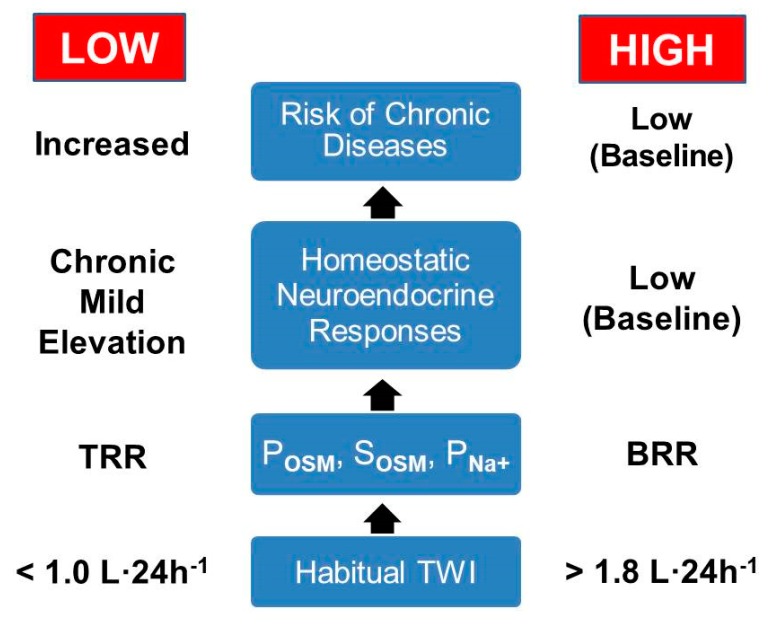
Proposed series of events that lead to the differential risk of chronic diseases in LOW and HIGH. Homeostatic neuroendocrine responses include increased plasma AVP and cortisol in LOW (see Section 5 above). Abbreviations: TRR, top half of the laboratory reference range; BRR, bottom half of the laboratory reference range [42]. This paradigm is based on eight source publications [7,9,18,42,61,90,91,92].

Figure 5 illustrates the proposed series of events which lead to a differential risk of chronic diseases for LOW and HIGH, as described in the previous three AVP-relevant paragraphs. This paradigm acknowledges that (a) a small increase of P_OSM_ stimulates AVP release into plasma (Figure 4), (b) sodium and its accompanying anions (e.g., Clˉ and HCO_3_^−^) comprise 90–95% of the osmotically active constituents of plasma [61,89] and (c) publications report a positive statistical association between S_Na+_ and multiple chronic diseases [42,90,91]. Thus, it is reasonable to ask if P_Na+_ or P_OSM_ is the decisive variable in this series of events. Two considerations inform this issue. First, it is likely that a considerable degree of inter-correlation exists among P_OSM_, P_Na+_ and the incidence of chronic diseases. Secondly, human experiments have examined the effects of administering various intravenous solutes on AVP release [92]. These trials determined that (a) P_OSM_ not specific solutes (e.g., Na^+^, mannitol, urea, glucose) mediate AVP release; (b) the osmoreceptor responds to both ionized and nonionized solutes; and (c) this mechanism involves some but not all solutes, because ineffective solutes cross the blood-brain barrier or are metabolized in the liver and other organs [52].

The current diet of many adults (e.g., characterized by an excess of animal protein and salt but insufficient in fruits, vegetables and water) does not optimally support human physiological and regulatory systems. Nevertheless, plasma electrolyte and clinical biomarkers can be maintained within a laboratory reference range, primarily by activating (i.e., often maximally) compensatory homeostatic mechanisms, even though an increased risk of multi-organ dysfunction and damage result from such chronic corrective homeostatic activities [93]. This suggests that reliance on laboratory reference ranges (i.e., the statistical prediction interval in which 95% of the population exist) to determine health status can be flawed [91]. Also, reliance on laboratory reference ranges explains why the unique characteristics of LOW (i.e., plasma AVP, U_VOL_, U_OSM_, U_SG_) and the TWI differences between LOW and HIGH previously were considered to be unimportant. To the contrary, Allen and colleagues [42] concluded that S_Na+_ levels in the upper half of the “normal range” should be treated as a clinical risk factor that prompts recommendation for modification of water and salt intake. Their conclusion suggests that not all “normal” laboratory blood values represent a similar risk of chronic disease [93]. Further, if this paradigm of disease risk (Figure 5) is verified, educational campaigns that are designed to increase water intake in healthy individuals, especially low volume consumers, will become important for modifying drinking habits and lower the risk of chronic diseases in LOW.

## 7. Future Research

### 7.1. Experimental Considerations

We recommend that the following three factors be considered during the design, control, data collection and interpretation of future investigations regarding the characteristics that distinguish LOW from HIGH (Table 1 and Table 2) and their attendant impact on disease risk. These hereditary, physiological and behavioral factors show that (a) the brain’s regulation of fluid-electrolyte homeostasis is dynamic and complex and (b) redundant mechanisms protect body water volume/tonicity and blood volume/pressure [94].

### 7.2. Variability of the P_OSM_ Threshold for Thirst

**Figure 6:**
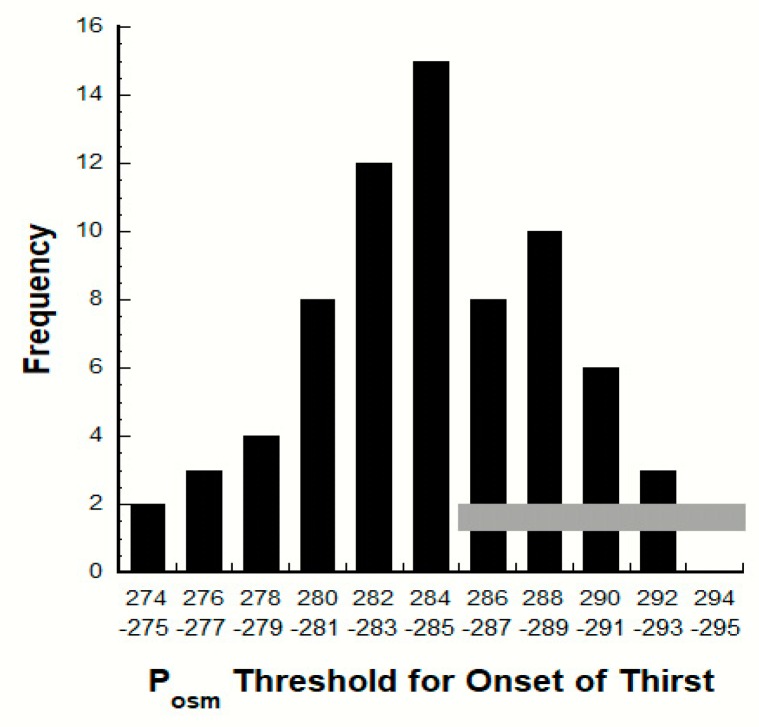
Frequency distribution of the plasma osmolality (P_OSM_) threshold for the onset of thirst. The horizontal gray zone represents the laboratory reference range of P_OSM_ values (285–295 mOsm·kg^−1^) for healthy adults [47].

Figure 6 presents a frequency distributions for the P_OSM_ threshold at which thirst is initially sensed. These data originally appeared in five human studies [53,54,61,92,95] that induced changes of blood osmolality and volume (e.g., via orthostasis, fluid deprivation, intravenous hypertonic saline infusion) during controlled laboratory experiments. The thirst threshold was defined in these studies as the axis intercept determined via linear regression equation. This frequency distribution (a) illustrates the great variability [96] that results from a complex, dynamic network of sensory nerves and neuroendocrine responses [1,4,97,98]; and (b) has one peak, usually interpreted as evidence of polygenic or multifactorial inheritance, similar to many other genetically influenced human traits [95].

Figure 6 provides insights into the nature of thirst in HIGH and LOW. For example, those adults whose thirst onset threshold is considerably below the laboratory reference range (e.g., P_OSM_ < 284 mOsm·kg^−1^, *n* = 17; [47]) are more likely to be chronically thirsty. This is one possible explanation for the greater thirst intensity (versus LOW, see above) that HIGH individuals experience. In contrast, other adults exhibit a P_OSM_ threshold for thirst of 295 mOsm·kg^−1^ (*n* = 17 in Figure 6). We recommend that these individuals not “drink to thirst” because they will sense thirst only when they are markedly dehydrated. Rather, they should take action to increase their own habitual TWI.

Although the five original investigations did not include TWI measurements or designate which test participants were HIGH or LOW [53,54,61,92,95], their findings suggest that differences of daily water intake (e.g., LOW and HIGH) are influenced by large biological variability. Figure 6 implies genetic variation that is relevant to habitual TWI. Assuming that all individuals in Figure 6 were within the laboratory reference range of healthy adults (285–295 mOsm·kg^−1^) at the time of testing, it is logical that those with a low P_OSM_ threshold for the onset of thirst are likely to be in the HIGH group (i.e., greater self-ratings of thirst; see Section 4.2 above) and those with a high thirst threshold are likely to be in the LOW group because they report lower thirst. This paradigm is consistent with the interpretation of Johnson et al. [18] who suggested that LOW and HIGH employ different homeostatic mechanisms because of their different habitual daily TWI. They concluded that HIGH maintains normal P_OSM_ and total body water predominantly via increased dependence on thirst and water consumption, whereas LOW maintains fluid-electrolyte homeostasis predominantly via neuroendocrine-controlled water conservation which includes elevated plasma AVP concentration [18]. However, the validity of this concept is worthy of further investigation. To our knowledge, no research has verified the plasticity or stability of an individual’s P_OSM_ threshold during a controlled intervention (e.g., modifying TWI) lasting weeks or months.

### 7.3. Renal Osmolar Excretion: Differences Due to Diet

When food and beverages are consumed and digested, the dietary osmolar content is processed in the gut and excess osmoles are eliminated in solid and liquid excreta. An influx of osmotically active particles enters the extracellular compartment during the subsequent minutes and hours. The stability of P_OSM_ and the contents of blood and urine, in the presence of this inward water and solute flux, are regulated by a sensitive brain osmoreceptor–AVP–renal response, as described above (Figure 4). 

The 24-h renal osmolar excretion (ROE) varies among individuals and across days due to differences of food mass, macronutrient composition and physical activity. A wide range of ROE (e.g., laboratory reference range, 283–1215 mOsm·24 h^−1^ for women) is possible in adults, as illustrated in Table 3. Because concentrated urine is a primary etiological factor in kidney stone formation and urinary tract infections [13,14,15,16,28,99,100], it is worthy of future studies that identify ways to simply and effectively reduce ROE.

### 7.4. Drinking Behavior & Fluid Composition

In addition to the P_OSM_ effects illustrated in Figure 6, the oropharyngeal region (e.g., drinking, dry mouth) influences plasma AVP non-osmotically via the central nervous system [103,104]. This response involves a rapid reduction of plasma AVP concentration soon after drinking, before P_OSM_ or plasma volume have changed. Oropharyngeal neural signals also rapidly reduce thirst and fluid intake [57,104] because they are integrated with volume/pressure/tonicity signals in the hypothalamus, then transferred to higher brain regions where drinking behavior is adjusted [105]. Hypothetically, HIGH could experience a more intense thirst and lower plasma AVP level than LOW (see above) because of inter-individual differences in the central processing of oropharyngeal stimuli or salivary flow rate [106]. To our knowledge, this non-osmotic hypothesis has not been tested and assumes that such variability is unimodal, similar to the frequency distributions shown in Figure 6.

The volume consumed, number and time elapsed between drinking events also may distinguish LOW from HIGH. When a large bolus of pure water or hypotonic fluid is consumed rapidly (e.g., 1.2 L in 4 min), this water enters the blood and the kidneys excrete a large volume of dilute urine (e.g., urine specific gravity of 1.005) to defend against fluid overload, before P_OSM_ changes and intracellular-extracellular fluids equilibrate fully [107]. This phenomenon occurs even when the host is dehydrated, when oropharyngeal signals to the brain decrease plasma AVP concentration rapidly (i.e., by 70%–85% within 3 min) and for a duration of at least 70 min [108]; this suggests that LOW could benefit (i.e., by decreasing plasma AVP chronically) from consuming fluids at regular intervals throughout the day. In this acute scenario, urine measurements mirror the volume of fluid consumed rather than a change of TBW [109]. Alternatively, when pure water or a hypotonic fluid is consumed slowly in small aliquots, the extracellular and intracellular compartments equilibrate gradually, P_OSM_ is not greatly perturbed and urine volume, specific gravity and osmolality are mildly altered [107,110]. These two scenarios (i.e., few large boluses versus many small aliquots) suggest that patterns of drinking throughout the day can influence plasma AVP [103,104] and thirst responses [111,112] and may distinguish LOW from HIGH. However, swallowing a larger volume (e.g., 0.8–1.1 L/3.5 min) inhibits AVP release and reduces the intensity of thirst more than sipping a small volume (e.g., 51–74 mL/3.5 min) when the number of swallowing events is controlled [111].

## 8. Nutritional Recommendations for LOW

### 8.1. Five Dietary and Hydration Goals

Considering the statistical and epidemiological associations of low daily water consumption and/or elevated plasma AVP with an increased risk of chronic diseases and reduced lifespan [7,28,29,30,31,32,33,34,35,36,37,38,39,40,42,43], simple and valid ideas to help LOW increase TWI are important [7,42,113] because habitual TWI is influenced by attitudes, learning and conditioning [114,115,116]. The following paragraphs provide dietary and hydration recommendations for LOW that have five goals—select solid foods with a high water content; take action to increase consumption of water and beverages; reduce daily dietary osmolar load by moderating specific foods (i.e., thereby reducing 24-h ROE and obligatory urine volume); self-assess hydration status; and avoid overdrinking. If utilized by LOW, these recommendations have potential to reduce P_OSM_, urine concentration and the osmolar load that the kidneys filter or excrete each day.

### 8.2. Select Solid Foods with a High Water Content

The portion of TWI that is derived from moisture in solid foods each day varies considerably across nations (mean range in the U.S.A., 17%–25%; mean in the U.K. 27%, France 36%, China 40%) and across individuals within specific demographic groups. For example, when fluid consumption data were divided into deciles, the percent of food moisture decreased from 44% (decile 1) to 30% (decile 10) in a French sample (n = 1,062 adults; median TWI, 1.8 L·24 h^−1^) and from 36% to 16% in a U.K. sample (*n* = 2,083 adults; median TWI, 2.2 L·24 h^−1^) [117]. Table 4 shows that the food moisture of LOW ranged from 31%–46% of TWI, whereas it ranged from 14%–22% in HIGH. The consistent trends within these data indicate that moisture in solid foods represents a meaningful portion of TWI for LOW that would be smaller if they habitually selected foods with a low water content. Recalling our proposal regarding the importance of maintaining a plasma AVP of < 2 pg·mL^−1^ (see Figure 2), a reasonable minimum daily TWI goal for adults is to consume more than 1.8 L·24 h^−1^ (Figure 2; [10,11]). To that end, Table 5 presents an assortment of foods and their respective water contents [118]. Selecting soups, fruit, vegetables and other water-rich foods can increase TWI more than 1.0 L·d^-1^; this approach also encourages a shift of nutrient intake toward a healthful plant-based diet that reduces cardiovascular disease risk [119,120]. However, food selection should include consideration of the salt content, especially that of processed foods, as described in Section 8.4.2 below.

Interestingly, in all data described in the previous paragraph, the absolute amount of moisture in food is similar (0.5–0.8 L·24 h^−1^) for LOW and HIGH and is smaller than the volume of water consumed in fluids (Table 4). This suggests that focusing on ways to increase consumption of water and beverages will have considerable impact on TWI. Therefore, we recommend that LOW take personal responsibility for prioritizing drinking behavior and take action to increase habitual TWI. This will involve cultivating personal awareness, setting TWI goals, organizing the work/home environment with reminders to drink and simplifying availability/access.

### 8.3. Act to Increase TWI

For LOW, increasing TWI and maintaining a low-normal P_OSM_ or S_OSM_ are important components of optimal health [7,41,42]. Increasing 24-h TWI can reduce or eliminate neuroendocrine responses (e.g., AVP, ANG II, aldosterone, cortisol; Figure 2) which counteract hypovolemia and elevated osmolality and reduce the risk of chronic diseases (Figure 5). Similarly, increasing TWI is important for elderly adults who live in geriatric facilities, because dehydration is associated with significant adverse outcomes in older people despite being largely preventable and treatable [121]. For example, two recent European studies published in *Nutrients* reported that dehydration (P_OSM_ > 295 mOsm·kg^−1^) was observed in 58.4% of 358 individuals (86% > 75 y) [122] and that a S_Na+_ value ≥ 140 mMol·L^−1^ could be used as a first-step screening procedure for detecting underhydration in geriatric patients [99]. Similar to LOW, older adults can be encouraged to increase TWI by building upon existing habitual drinking patterns [121]. 

Therefore, we offer the following 6 simple actions because we anticipate that they will help to increase the daily intake of water and beverages by LOW and the elderly. (1) Select fluid flavors, colors and temperatures that are pleasurable while considering energy and sugar contents. (2) Place a water bottle next to your computer or wear a refillable bottle on your belt. Refill the bottle each time you empty it. (3) Develop a habit of drinking a glass of water when you wake, before each meal, after you visit the toilet or when you are waiting for someone or an event. (4) Find a water scorekeeping app online or maintain a paper diary/checklist, to record the number of glasses you consume. Consider using a container marked with volume indicators or measure the volume of frequently used containers to accurately assess intake. (5) Set a personal water intake goal for each segment of the day. (6) A dietitian can help you to determine a TWI goal based on the number of calories you consume each day (1.0–1.5 mL·kcal^−1^ of food energy) [100,123].

### 8.4. Reduce 24-h Osmolar Load

Concentrated urine has been acknowledged in the preceding paragraphs as a primary etiological factor in multiple kidney disorders, including kidney stone formation and urinary tract infections [13,14,15,16,28,124,125]. In addition to increasing TWI (the solvent) as a means to reduce P_OSM_ and the renal osmolar load, dietary osmolar content (the solute) also can be modified by reducing consumption of specific foods or additives.

**Figure 7:**
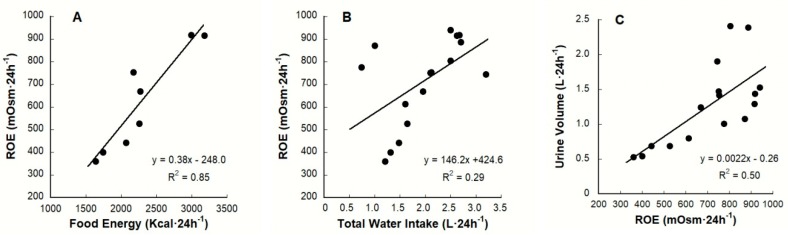
Relationships between 24-h renal osmolar excretion (ROE) and food energy, total water intake and urine volume. Each symbol represents a group mean (*n* = 14–682) from the six studies in Table 3. (**A**): the strong relationship between food energy and ROE; (**B**): the weak relationship between TWI and ROE; (**C**): as ROE increases, the obligatory urine volume increases.

Figure 7 presents data from six studies that are relevant to ROE and TWI [9,18,20,21,101,102]. Panel A illustrates the strong influence of dietary osmolar load on ROE. Although wastes and the products of metabolism contribute to extracellular osmolality (P_OSM_ and S_OSM_), dietary energy content (Kcal) accounts for 85% of the variance in ROE. Thus, Panel A supports the concept that renal osmolar load each day is largely influenced by diet [93,101,126,127,128] and agrees with the dietary modification plan advanced by Amro and colleagues in 2016 (i.e., which led to reduced plasma AVP and a reduced TWI requirement for AVP reduction in kidney disease patients) [128]. In contrast, the correlation between TWI and ROE (Figure 7, Panel B) is weaker (R^2^ = 0.29). It also is relevant that the mean ROE of LOW (0.7–1.6 L·24 h^−1^ TWI) may be either less than or greater than the ROE of HIGH (2.5–3.2 L·24 h^−1^ TWI) as shown in rows 13–18 of Table 3; we ascribe these heterogeneous data to differences of the osmolar content of the diets consumed by LOW and HIGH during controlled intervention studies [9,18,20,21].

#### 8.4.1. Dietary Protein

The influence of dietary protein on ROE is evident in a published case report involving a female patient (40 y age, 65 kg body mass, 20 kg·m^−2^ body mass index) who processed a large daily renal solute load of 1630 mOsm·24 h^−1^ (compare to column 1 in Table 3) during her initial clinical evaluation for polyuria [129]. She consumed a high total protein content (133 g protein·d^−1^, 2.0 g·kg^−1^·d^−1^), largely due to nutritional supplements; each meal included 4 oz of meat, a commercial protein shake (30 g) and a protein supplement bar (20 g). The accompanying water intake was > 7.5 L·d^−1^ and her U_VOL_ ranged from 6.5–9.6 L·d^−1^ (see Table 1 to compare these values to LOW and HIGH). During the course of diet-based therapy, this patient was advised to reduce daily intake of protein from 2.0 to 1.0 g·kg^−1^, sodium to 2759 mg·d^−1^ (120 mEq·d^−1^) and potassium to 3910 mg·d^−1^ (100 mEq·d^−1^). As a result, her ROE decreased from 1630 (pre-treatment) to 1077 and 600 mOsm·24 h^−1^, respectively, at 2- and 3-week follow-up examinations. Her U_VOL_ concurrently decreased to 3.5 L·24 h^−1^ [129], indicating a strong relationship between renal solute load and U_VOL_. 

Panel C of Figure 7 illustrates the obligatory urine volume necessary to excrete the total osmolar load presented to the kidneys during a 24-h period [101,126,128]. In humans, the two largest components of renal solute load are urea (i.e., a nitrogenous product of protein digestion) and salt [126,127,128]. Increasing the 24-h osmolar load (e.g., by increasing the amount of dietary protein and salt) increases ROE and the obligatory urine volume [129,130]. As an example, a randomized, controlled, crossover dietary intervention study evaluated the effects of dietary protein intake on the hydration status of free-living adults [127]. Male test participants consumed 3 different eucaloric diets containing 0.8 (low), 1.8 (moderate) and 3.6 (high) g of protein·kg^-1^·d^-1^ for 4 weeks each. Subjects were fed all meals (i.e., weighed and portioned to meet each protein level) at a university nutrition facility. The step-wise increase of ROE across the three diets resulted in significantly different (*p* < 0.05) urine specific gravity and blood urea nitrogen values. P_OSM_ at the end of each 4-week treatment rose with increasing protein load (*p* < 0.05), in a dose-response manner (low, 277; moderate, 282; high, 287 mOsm·kg^−1^). Considering the exacting sensitivity of plasma AVP regulation (see panel B, Figure 4 and Section 6 above), these experimentally-induced P_OSM_ differences (i.e., due to increased plasma urea) likely stimulated plasma AVP (not measured) to be elevated during the 1.8 and 3.6 g protein·kg^−1^·d^−1^ diets. 

Both animal and human studies support adequate-to-moderate protein consumption. In fact, these studies demonstrate that the altered renal function and morphological changes which occur during a chronic high protein diet are strictly dependent on plasma AVP [131]. The human experiments [132] evaluated the effects of low (0.55 g protein·kg^−1^·d^−1^) versus high (2.0 g protein·kg^−1^·d^−1^) protein diets (randomized, within-subject, crossover design) on the fluid-electrolyte regulatory hormones AVP, renin and aldosterone across 4 days. All meals were prepared and eaten in a medical school clinical research center. While consuming the high protein diet, the three plasma hormones were significantly elevated (*p* < 0.05; fasted state) on the morning of day 5. Interestingly, the mean plasma AVP concentration during the low protein diet (1.65 pg·mL^−1^) was below the 2.0 pg·mL^−1^ threshold shown in Figure 2 but was well above this threshold (4.33 pg·mL^−1^) during the high protein diet. In total, these findings [127,131,132] and others [133] suggest that adults who habitually consume high protein diets experience chronically elevated plasma AVP and pathophysiological changes [28]. Although not yet verified in clinical trials or laboratory experiments, it is possible that the detrimental effects of a chronic high protein diet combined with a habitually low TWI (i.e., due to elevated plasma AVP) are additive.

#### 8.4.2. Dietary Salt

The 24-h osmolar load processed by the kidneys is not totally due to dietary protein; NaCl also is an important contributor. One published report indicated that adult 24-h osmolar excretion consists of 35% NaCl, 35% urea nitrogen (i.e., from protein metabolism) and 30% uric acid, amino acids, creatinine and assorted anions or cations [130]. A second study stated that NaCl contributes 35%–44% and urea nitrogen approximately 40% of total renal solute excretion [126]. A third publication expressed ROE as 35% additives (e.g., NaCl), 25% urea and 40% food [128]. Finally, Bhasin and associates [129] calculated ROE as 20%–32% Na^+^, 50–58% urea nitrogen and 18%–22% K^+^. This suggests that adults who habitually consume high NaCl diets may experience a minor P_OSM_ increase and chronically elevated plasma AVP, similar to LOW, because (a) sodium and its accompanying anions (e.g., NaCl, NaHCO_3_) account for 90%–95% of P_OSM_, (b) secretion of AVP is very responsive to small increases of P_NA+_ as it is to P_OSM_ [92] and (c) research suggests that a large oral intake of salty food will induce AVP secretion that favors water conservation at the expense of a limited ability to excrete sodium [134].

The U.S. National Academy of Sciences has recognized a disease risk reduction level [135], defined as the lowest level of sodium intake for which there is sufficient strength of evidence to characterize a chronic disease risk reduction for cardiovascular disease and hypertension. For men and women of all ages, this quantity is 2300 mg Na^+^ (5.8 g NaCl, ~ 1 teaspoon) per day and serves LOW and HIGH as a meaningful goal for daily sodium intake. However, in the United States, 97% of men (> 19 years) and 80% of women (> 19 years) exceed this amount (Table 1–6 [135]). In 45 countries, adults consume an average of 3666 (range 2622–4830) mg Na^+^ (9.3 g NaCl) each day [136].

Single food items can contribute greatly to salt over-consumption (Table 5). To moderate total osmolar load on the kidneys, we recommend that LOW avoid the following foods which contain a high sodium content [118,137]—bacon, center cut, 3 slices (5409 mg Na^+^); onion soup, dry mix, 1 packet (3132 mg); table salt, 1 teaspoon (2300 mg); tomato sauce, canned, 1 cup (1482 mg); breakfast sausage biscuits, fast food (1080 mg); clam chowder, 1 cup (992 mg); cottage cheese, 1 cup (850 mg); chicken and rice soup, 1 cup (815 mg); macaroni and cheese, 1 cup (720 mg); and 2 hot dogs, packaged (713 mg). Processed foods (e.g., those which contain NaCl for flavoring or preservation) and restaurant meals often are sources of large amounts of sodium. Interestingly, observational studies indicate that salt added by the consumer at the table accounts for only about 5%–6% of NaCl consumed. This evidence suggests that reductions may be best achieved by reducing the NaCl added to commercially-processed and restaurant-prepared foods, while heeding the current dietary guidelines to reduce the discretionary addition of salt during meals [138,139]. However, increased energy intake or altered consumption of processed/restaurant foods likely will alter the percentage contribution of NaCl added by the consumer during meals.

### 8.5. Self-assess Hydration Status

By virtue of habitual low water intake, LOW individuals are predisposed to elevated plasma AVP, making it valuable for them to routinely self-assess hydration status as a guide to water and beverage intake. However, more than a dozen hydration assessment techniques exist and no single method is valid in all scenarios and for all individuals [46,140]. Because some of these techniques require laboratory instruments or technical expertise (e.g., P_OSM_ and P_Na+_ measurements) and because most adults cannot utilize these methods during daily activities, hydration assessment techniques should be simple, non-technical, accurate, safe and inexpensive [45]. We recommend four simple techniques—body weight, thirst, void number and urine color [2,25,46,140]. First, if morning body weight is recorded upon waking using an accurate digital floor scale (± 0.1 kg precision), an average or typical weight can be determined within a few days [141]. Using this baseline, a weight loss of 0.5 kg (1 lb) indicates that additional water should be consumed above the usual TWI. Second, thirst is initially sensed when a 1%–2% body weight loss occurs and increases with greater dehydration. The morning sensation of thirst upon waking is a strong predictor of dehydration [142]. Third, the number of bladder voids per day can be used to estimate hydration status. Two published reports, observing healthy young adults during daily activities, indicated that euhydrated individuals visited the toilet more often (*p* < 0.001) than hypohydrated [143,144]. Fourth, urine color is sensitive to dehydration and is responsive to changes of fluid intake [145]. As whole-body dehydration develops, urine color darkens because kidneys are reabsorbing/conserving water. Pale yellow or straw colored urine indicates a state of euhydration [46]. A validated 8-point urine color chart [9,145,146] is available at www.hydrationcheck.com. Because the interpretation of hydration status varies across a typical day, depending on which technique is used, it is wise to self-assess hydration status using two or more of these techniques [25].

### 8.6. Avoid Overdrinking

Our recommendation for LOW to increase TWI should not be interpreted as a call to drink excessively. Although not common, it is possible to become critically ill by drinking too much water. In contrast to the mildly increased P_OSM_ and P_Na+_ which LOW experiences, overdrinking can result in hyponatremia and hyposmolality resulting from dilution of body fluids. Known as water intoxication, this medical disorder requires emergency medical care when acute and severe [90]. Without continuous access to laboratory tests, we recommend that LOW utilize urine color, void frequency and Adequate Intakes to avoid overdrinking. Adequate Intakes for water have been published on the basis of age and sex by the European Food Safety Authority (women 2.0 and men 2.5 L·24 h^−1^ [10]) and the U.S. National Academy of Medicine (women 2.7 and men 3.7 L·24 h^−1^ [11]). These values provide safe daily intake goals that are expressed as TWI (water + beverages + food moisture) and assume that approximately 20% of TWI is consumed as food moisture.

Urine color self-assessment provides useful feedback regarding overdrinking. Among urine samples collected across 12 days (n = 290 samples [48]), less than 5% had a urine color of pale yellow and no samples were colorless. Simply stated, only 3%–5% of all specimens are very dilute in men [48] and women [49]. Therefore, 2–4 consecutive urine samples that appear pale yellow or colorless (i.e., indicating that the kidneys are releasing excess water) likely indicate overdrinking. 

## 9. Conclusions

The present review article describes characteristic differences between LOW and HIGH, an increased risk of several chronic diseases and reduced lifespan for LOW and proposes a theoretical model of differential chronic disease risk. This model is based upon homeostatic neuroendocrine responses (i.e., increased plasma AVP and cortisol in LOW) which are sensitive to a small difference of 24-h TWI, or a small change of P_OSM_ and provides testable hypotheses for future prospective studies and randomized controlled trials. If this chronic disease model is verified in future clinical investigations, educational campaigns aimed at increasing TWI will become important for modifying the drinking habits of LOW and preventing chronic diseases.

## Figures and Tables

**Table 1:** Blood and urine characteristics of healthy low volume (LOW) and high volume (HIGH) drinkers.

Variable (unit)	Baseline Mean Values		Statistical Significance: Low Versus HIGH	Laboratory Reference Ranges for Healthy Adults ^a^	References
	LOW	HIGH			
**TWI (L·24 h^−1^)**	**0.74**	**2.70**	IV		[9] ^b^
P_OSM_ (mOsm·kg^−1^)	289 ^+^	288 ^+^	NS	285–295	
P_AVP_ (pmol·L^−1^)	2.4 ^+^	1.5 ^+^	*p* < 0.001	0.3–3.3	
Hematocrit (%)	41.2	40.8	NS	41–53 ♂, 36–46 ♀	
P_CORT_ (nmol·L^−1^)	545	459	*p* = 0.012	138–690	
U_VOL_ (L) ^c^	1.01	2.39	*p* < 0.001	0.68–3.00 ♂, 1.17–2.41 ♀	
U_OSM_ (mOsm·kg^−1^) ^c^	767	371	*p* < 0.001	300–900	
U_SG_^c^	1.023 ^+^	1.010 ^+^	*p* < 0.001	1.001–1.035	
TWI (L·24 h^−1^)	1.0	2.5	IV		[20] ^d^
P_OSM_ (mOsm·kg^−1^)	292, 291 ^e^	289, 287 ^e^	NS	285–295	
U_VOL_ (L) ^c^	1.08, 0.97 ^e^	2.41, 2.48 ^e^	§	0.68–3.00 ♂, 1.17–2.41 ♀	
U_OSM_ (mOsm·kg^−1^) ^c^	807, 875 ^e^	334, 331 ^e^	§	300–900	
U_SG_^c^	1.021, 1.022 ^e^	1.010, 1.009 ^e^	§	1.001–1.035	
TWI (L·24 h^−1^)	1.6	3.2	IV		[18,21] ^f^
S_OSM_ (mOsm·kg^−1^)	295	293	NS	285–295	
P_AVP_ (pg·mL^−1^)	2.7 ^+^	1.3 ^+^	*p* < 0.001	0.3–3.3	
Hematocrit (%)	42	41	NS	41–53 ♂, 36–46 ♀	
S_Na+_ (mmol·L^−1^)	142	141	NS	136–145	
TPP (g·dL^−1^)	7.3	7.0	*p* < 0.05	5.5–8.0	
U_VOL_ (L) ^c^	0.8	1.9	*p* < 0.05	0.68–3.00 ♂, 1.17–2.41 ♀	
U_OSM_ (mOsm·kg^−1^) ^c^	766	392	*p* < 0.05	300–900	
U_SG_^c^	1.021	1.011	*p* < 0.05	1.001–1.035	

Note: all values in columns 2 and 3 are within the laboratory reference ranges shown in column 5. ^a^, based on three source publications [47,48,49]; ^b^, LOW(*n* = 39, 46% ♀) and HIGH (*n* = 32, 69% ♀); ^c^, measured in 24-h urine samples; ^d^, LOW (*n* = 30, 63% ♀) and HIGH (*n* = 22, 100% ♀); ^e^, two baseline days were reported; ^f^, LOW (*n* = 14, 100% ♀) and HIGH (*n* = 14, 100% ♀). ^+^, median values. §, not reported or data unavailable. Abbreviations: TWI, total water intake (water + beverages + food moisture); IV, independent variable that was experimentally controlled; P_OSM_, plasma osmolality; NS, not significant at *p* < 0.05; P_AVP_, plasma arginine vasopressin; P_CORT_, plasma cortisol concentration; U_VOL_, urine volume; U_OSM_, urine osmolality; U_SG_, urine specific gravity; S_OSM_, serum osmolality; S_Na+_, serum sodium; TPP, total plasma protein.

**Table 2:** Physiological changes that occurred when low volume (LOW) and high volume (HIGH) drinkers reversed their habitual 24-h total water intake (TWI) for 3–4 days. Baseline (pre-intervention) values appear in Table 1.

Variable (unit)	Days of Modified 24-h TWI Intervention	Physiological Changes After 24 h TWI Was Modified		Authors
		LOW → HIGH	HIGH → LOW	
P_OSM_ (mOsm·kg^−1^)	3 ^a^	0	+3	[20] ^b^
U_VOL_ (L) ^c^	3 ^a^	+1.355	+1.450	
U_OSM_ (mOsm·kg^−1^) ^c^	3 ^a^	−486	+417	
U_SG_^c^	3 ^a^	−0.012	+0.010	
S_OSM_ (mOsm·kg^−1^)	1–4 ^d^	−2	+1	[18,21] ^e^
Hematocrit (%)	1–4 ^d^	0	+1.0	
TPP (mg·dL^−1^)	1–4 ^d^	+0.2	−0.1	
P_AVP_ (pg·mL^−1^)	1–4 ^d^	−1.2	+1.3	
S_Na+_ (mmol·L^−1^)	1–4 ^d^	0	+1	
U_VOL_ (L) ^c^	1–4 ^d^	+1.4	−0.7	
U_OSM_ (mOsm·kg^−1^) ^c^	1–4 ^d^	−492	+201	
U_SG_^c^	1–4 ^d^	−0.013	+0.006	

^a^, third intervention day; ^b^, modified TWI: LOW, from 1.0 at baseline to 2.5 L·d^−1^ during controlled intervention and HIGH from 2.5 at baseline to 1.0 L·d^−1^; ^c^, measured in 24-h urine samples; ^d^, change values (columns 3 & 4) are means of 4 observation days; ^e^, modified TWI: LOW, from 1.6 at baseline to 3.5 L·d^−1^ during controlled intervention and HIGH from 3.2 at baseline to 2.0 L·d^−1^. Abbreviations are identical to those in Table 1.

**Table 3:** Renal osmolar excretion (ROE) in 24-h urine samples of adults and children.

Mean ROE (mOsm·24 h^−1^) ^a^	Study Participants (n)	Characteristics	References
401	Boys (*n* = 189)	4–6.9 y	[101]
527	Boys (*n* = 174)	7–10.9 y	
359	Girls (*n* = 181)	4–6.9 y	
443	Girls (*n* = 174)	7-–10.9 y	
941	Men (*n* = 507)	Mean age, 47 y	
752 ^b^	Women (*n* = 682)	Mean age, 43 y	
669	Women (*n* = 101)	18–24 y	[102]
754	Women (*n* = 468)	25–49 y	
915	Men (*n* = 70)	18–24 y	
918	Men (*n* = 308)	25–49 y	
656–1,222	Men (*n* = 639)	Reference Range ^c^	
283–1,215	Women (*n* = 889)	Reference Range ^c^	
775	Test subjects (22♂, 17♀)	LOW consuming 0.74 L·24 h^−1^; mean age, 31 y	[9] ^d^
887	Test subjects (10♂, 20♀)	HIGH consuming 2.70 L·24 h^−1^; mean age, 32 y	
872	Test subjects (11♂, 19♀)	LOW consuming 1.0 L·24 h^−1^; mean age, 25 y	[20] ^d^
805	Test subjects (22♀)	HIGH consuming 2.5 L·24 h^−1^; mean age, 25 y	
613	Test subjects (14♀)	LOW consuming 1.6 L·24 h^−1^; mean age, 20 y	[18,21] ^d^
745	Test subjects (14♀)	HIGH consuming 3.2 L·24 h^−1^; mean age, 21 y	

^a^, ROE (mOsm·24 h^−1^) = urine volume (L·24 h^−1^) x urine concentration (mOsm·kg^−1^) in a 24-h sample, assuming that 1 L corresponds to 1 kg; ^b^, median value for individuals of all ages; ^c^, the statistical prediction interval in which 95% of the population exists (> 18 y of age); ^d^, supporting data appears in Table 1 above.

**Table 4:** Dietary water sources of habitual low volume (LOW) and high volume (HIGH) drinkers in two investigations.

Avenues of Water. Gain & Loss (L·24 h^−1^ or kg·24 h^−1^)	LOW	HIGH	References
Fluids Consumed ^a,b^	0.74	2.70	[9]
Moisture in food ^b^	0.64 ^c^	0.78 ^c^	
24-h Total Water Intake ^d^	1.38	3.48	
Percent of TWI from moisture in food	46	22	
Fluids Consumed ^a,b^	1.1	2.5	[18]
Moisture in food ^b^	0.5	0.7	
24-h Total Water Intake ^d^	1.6	3.2	
Percent of TWI from moisture in food	31	22	

^a^, plain water + beverages; ^b^, assessed via paper diet records or electronic food questionnaire; ^c^, unpublished mean data obtained from authors, measured on consecutive days; ^d^, sum of moisture content of food + total water volume in beverages. Note: the number of test participants in each study appears in Table 3.

**Table 5:** Water, energy, protein and sodium contents of common foods [118].

Food Item	Serving Size and Weight (g)	Water (%)	Energy (Kcal)	Protein (g)	Sodium (mg)
**Dairy Products**					
Cottage Cheese	1 cup (210)	79	217	26	850
American Cheese	1 oz (28)	39	106	6	406
Swiss Cheese	1 oz (28)	42	95	7	388
Frozen Yogurt	½ cup (72)	64	115	3	71
Ice Cream	½ cup (66)	61	133	2	53
Milk (Whole)	1 cup (244)	88	150	8	120
Milk (2%)	1 cup (244)	90	102	8	122
Yogurt (Fruited)	8 oz (227)	74	231	10	133
Yogurt (Plain)	8 oz (227)	85	144	12	159
**Eggs**					
Hard boiled	1 Large (50)	75	78	6	62
Scrambled	1 Large (61)	73	101	7	171
**Fats and Oils**					
Butter (salted)	1 stick (113)	16	813	1	937
Margarine (soft)	1 cup (227)	16	1626	2	2449
Canola Oil	1 cup (218)	0	1927	0	0
Olive Oil	1 cup (216)	0	1909	0	0
French Dressing	1 Tbsp (16)	38	67	Trace	214
Italian Dressing	1 Tbsp (15)	38	69	Trace	116
Mayonnaise	1 Tbsp (14)	15	99	Trace	78
**Fish**					
Baked Haddock	3 oz (85)	74	95	21	74
Baked Salmon	3 oz (85)	62	184	23	56
Scallops	3 oz (85)	73	95	20	225
Shrimp (canned)	3 oz (85)	73	102	20	144
Tuna (water pk.)	3 oz (85)	73	109	20	320
**Fruits and Fruit Juices**					
Apple (Raw)	1 medium (138)	84	81	Trace	0
Apple Juice	1 cup (248)	88	117	Trace	7
Applesauce	1 cup (244)	88	105	Trace	5
Pear (Raw)	1 medium (122)	88	51	1	0
Avocado	1 oz (28)	80	32	Trace	3
Banana (Raw)	1 medium (118)	74	109	1	1
Cherries (Raw)	10 cherries (68)	81	49	1	0
Grapefruit (Raw)	½ grapefruit (123)	91	37	1	0
Green Grapes	10 grapes (50)	81	36	Trace	1
Mango	1 cup (165)	82	107	1	3
Cantaloupe	1 cup (160)	90	56	1	14
Honeydew	1 cup (170)	90	60	1	17
Orange (Raw)	1 medium (131)	87	62	1	0
Orange Juice	1 cup (248)	88	112	2	2
Peach (Raw)	1 medium (98)	88	73	1	0
Pineapple	1 cup (155)	87	76	1	2
Plum (Raw)	1 medium (66)	85	36	1	0
Raspberries	1 cup (123)	87	60	1	0
Strawberries	1 cup (166)	92	50	1	2
Watermelon	1 cup (152)	92	49	1	3
**Grain Products**					
Corn Grits	1 cup (242)	85	145	3	0
Cream of Wheat	1 cup (251)	87	133	4	3
Macaroni	1 cup cooked (140)	66	197	7	1
Egg Noodles	1 cup cooked (160)	69	213	8	11
Oat Bran	1 cup cooked (219)	84	88	7	2
White Rice	1 cup cooked (158)	68	205	4	2
Spaghetti	1 cup cooked (140)	66	197	7	1
**Legumes, Nuts, Beans**					
Black Beans	1 cup cooked (172)	66	227	15	2
Red Kidney Beans	1 cup cooked (177)	67	225	15	4
Lima Beans	1 cup cooked (188)	70	216	15	4
Pinto Beans	1 cup cooked (171)	64	234	14	3
Hummus	1 Tbsp (14)	67	23	1	53
Peanut Butter	1 Tbsp (16)	1	95	4	75
**Soups, Sauces, Gravies**					
Clam Chowder	1 cup (248)	85	164	9	992
Tomato Soup	1 cup (248)	85	161	6	744
Chicken & Rice Soup	1 cup (241)	94	60	4	815
Lentil Soup	1 cup (242)	88	126	8	443
Minestrone Soup	1 cup (241)	87	123	5	470
Vegetable Soup	1 cup (238)	91	81	4	466
Turkey Gravy	¼ cup (60)	89	31	2	346
**Sugar and Sweets**					
Fruit Juice Bar	1 solid bar (77)	78	63	1	3
Gelatin	½ cup (135)	85	80	2	57
Vanilla Pudding	½ cup (142)	75	148	4	406
Tapioca	½ cup (113)	74	134	2	180
**Vegetables**					
Asparagus	1 cup cooked (180)	92	43	5	20
Green Beans	1 cup cooked (125)	89	44	2	4
Beets	1 cup cooked (170)	87	75	3	131
Broccoli	1 cup cooked (156)	91	44	5	41
Cabbage	1 cup cooked (150)	94	33	2	12
Carrots	1 cup cooked (156)	87	70	2	103
Cauliflower	1 cup cooked (124)	93	29	2	19
Celery	1 stalk (40)	95	6	Trace	35
Corn	1 cup cooked (164)	77	131	5	8
Cucumber	1 cup peeled (119)	96	14	1	2
Kale	1 cup cooked (130)	91	36	2	30
Mushrooms	1 cup cooked (156)	91	42	3	3
Okra	1 cup cooked (160)	90	51	3	8
Onion (Raw)	1 cup (160)	90	61	2	5
Green Peas	1 cup cooked (160)	89	67	5	6
Green Pepper	1 cup raw (149)	92	40	1	3
Potato	1 baked (202)	71	220	5	16
Spinach	1 cup cooked (180)	91	41	5	126
Summer Squash	1 cup cooked (180)	94	36	2	2
Butternut Squash	1 cup cooked (240)	88	99	3	5
Tomato (Raw)	1 cup (180)	99	38	2	16
Tomato Sauce	1 cup (245)	89	74	3	1482
